# Multisite Chronic Pain Accelerates Cognitive Decline Particularly in APOE e4 Allele Carriers

**DOI:** 10.1002/alz.091576

**Published:** 2025-01-09

**Authors:** Tyler R. Bell, Carol E. Franz, Imanuel Lerman, Christine Fennema‐Notestine, Matthew S. Panizzon, David A. Bennett, Jeremy A. Elman, William S. Kremen

**Affiliations:** ^1^ University of California, San Diego, La Jolla, CA USA; ^2^ University of San Diego California, San Diego, CA USA; ^3^ Department of Neurological Sciences, Rush Medical College, Chicago, IL USA

## Abstract

**Background:**

Chronic pain is a predictor of cognitive decline and dementia, but whether risk is related to a specific site of pain versus multisite pain is unclear. Multisite chronic pain is theorized to involve hyper‐excitability in pain receptors and pain‐processing brain regions, possibly caused by neuroinflammation. These factors may increase vulnerability to cognitive decline, particularly in those at elevated risk for Alzheimer’s disease. We examined how multisite chronic pain relates to cognitive decline in older adults, the possible moderating role of APOE‐ε4 allele status, and—in a postmortem subsample—associations with amyloid and tau.

**Method:**

We examined 751 participants without dementia at baseline from the Religious Orders Study/Rush Memory and Aging Project (ROSMAP; Mean age = 79.47, SD = 6.93, Mean education = 6.42, SD = 3.63; 64% women) followed for up to 30 years with repeated cognitive testing measuring episodic memory, working memory, perceptual orientation, and processing speed (Mean years followed = 7.32, SD = 4.51). Individuals with multisite chronic pain, defined as joint pain in >2 body regions over the first 2 study waves, were compared to those without pain. Amyloid‐beta and tau were measured in a postmortem subsample assessed around 10 months after their last clinical evaluation using computer‐assisted immunoreactivity assays across 8 regions associated with Alzheimer’s disease (hippocampus; entorhinal cortex; midfrontal cortex; inferior temporal; angular gyrus; calcarine cortex; anterior cingulate cortex; superior frontal cortex

**Result:**

Multisite chronic pain (n = 105) was associated with accelerated decline in episodic memory (b = ‐.03, p = .016) and processing speed (b = ‐.06, p<.001), particularly in APOE‐ε4‐positive individuals (bs = ‐.05 and ‐.04, respectively, ps<.01). Only APOE‐ε4‐positive individuals with multisite chronic pain showed significant decline in perceptual orientation (b = ‐.08, p = .015). Only APOE‐ε4‐positive individuals with multisite chronic pain had higher levels of amyloid‐beta load compared to those without chronic pain (b = .51, p<.001). No differences in tau burden were detected. People with single‐site chronic pain showed no significant differences from people without chronic pain.

**Conclusion:**

The results suggest that multisite chronic pain exacerbates cognitive decline, particularly in those at genetic risk for Alzheimer’s disease. In addition, the postmortem analyses suggest that early treatment of multisite chronic pain—a modifiable risk factor—might help to reduce amyloid accumulation.

